# Overexpression of the HECT ubiquitin ligase PfUT prolongs the intraerythrocytic cycle and reduces invasion efficiency of *Plasmodium falciparum*

**DOI:** 10.1038/s41598-019-54854-z

**Published:** 2019-12-04

**Authors:** Monika Jankowska-Döllken, Cecilia P. Sanchez, Marek Cyrklaff, Michael Lanzer

**Affiliations:** Center of Infectious Diseases, Parasitology, Heidelberg University Hospital, Im Neuenheimer Feld 324, 69120 Heidelberg, Germany

**Keywords:** Cell growth, Parasite development

## Abstract

The glms ribozyme system has been used as an amenable tool to conditionally control expression of genes of interest. It is generally assumed that insertion of the ribozyme sequence does not affect expression of the targeted gene in the absence of the inducer glucosamine-6-phosphate, although experimental support for this assumption is scarce. Here, we report the unexpected finding that integration of the glms ribozyme sequence in the 3′ untranslated region of a gene encoding a HECT E3 ubiquitin ligase, termed *Plasmodium falciparum* ubiquitin transferase (PfUT), increased steady state RNA and protein levels 2.5-fold in the human malaria parasite *P. falciparum*. Overexpression of *pfut* resulted in an S/M phase-associated lengthening of the parasite’s intraerythrocytic developmental cycle and a reduced merozoite invasion efficiency. The addition of glucosamine partially restored the wild type phenotype. Our study suggests a role of PfUT in controlling cell cycle progression and merozoite invasion. Our study further raises awareness regarding unexpected effects on gene expression when inserting the glms ribozyme sequence into a gene locus.

## Introduction

Many proteins are post-translationally modified by the covalent attachment of a small polypeptide, termed ubiquitin. Ubiquitination serves versatile biological purposes and can earmark a protein for degradation via the proteasome, regulate its enzymatic activity or interaction with other factors, or modulate its intracellular sorting and trafficking route^1–5^. Ubiquitination is catalyzed by a cascade of enzymatic reactions in which ubiquitin is activated, conjugated and finally ligated to the substrate protein^6^.

The human malaria parasite *P. falciparum* encodes 8 ubiquitin-activating enzymes (E1s), 14 ubiquitin-conjugating enzymes (E2s), and 54 ubiquitin ligases (E3s)^7^. The role of these enzymes in the biology and pathology of *P. falciparum* is only partly understood. For instance, UBA1 (E1), UBC7 (E2) and HRD1 (E3) were identified as major components of the endoplasmic reticulum-associated degradation (ERAD) pathway and were found to be essential^8^. In addition, *P. falciparum* maintains an ERAD-like ubiquitination pathway in the apicoplast, involving PfsUBA1 (E1), PfE2_Ap_ (E2) and PfE3c_Ap_ (E3), which are required for protein import into this organelle^9,10^. Furthermore, polymorphisms in two E3 ubiquitin ligases have been associated with reduced susceptibility to the antimalarial drugs pyrimethamine and artemisinin^11,12^. Other studies have implicated polymorphisms in deubiquitinating enzymes in altered responsiveness to chloroquine and artemisinin derivatives^13,14^.

We have recently associated polymorphisms in a HECT (homologous to E6AP C-terminus) E3 ubiquitin ligase, termed PfUT (MAL7P1.19 or PF3D7_0704600), with altered responsiveness to the antimalarial drug quinine and its enantiomer quinidine^15^. Apart from this report, very little is known about the biological function of this protein. PfUT shares some sequence homologies with the HECT ubiquitin-protein ligase UFD4 of *Saccharomyces cerevisiae*. UFD4 is a component of the ubiquitin fusion degradation pathway and it is also involved in the proteasome-mediated Arg/N-end rule pathway by polyubiquitinating proteins earmarked for degradation^16^. PfUT is a large transmembrane protein of 460 kDa localized at the ER/Golgi complex where it seems to associate with other factors forming a mega Dalton complex^15^. PfUT contains several structural features, including four predicted transmembrane domains, a catalytically active HECT domain and two long stretches of armadillo (ARM) repeats present in the cytoplasmic C- and N-terminal domains of the protein, respectively. Armadillo repeats typically mediate protein-protein interactions and might play a role in substrate recognition^17^. PfUT is post-translationally modified by phosphorylation at Y347, T372, S411 and S1873^18^ and by acetylation at K2929^19^ (Supplementary Fig. 1). According to the transcription profile^20,21^, *pfut* is relatively equally expressed throughout the intraerythrocytic cycle, with a slight decrease in late schizonts and merozoites.

Gene disruption studies have provided conflicting results regarding the importance of *pfut* in parasite survival. While a study conducted in the mouse malaria model system *P. berghei*, suggested an essential role of the *pfut* orthologue in parasite biology^22^, another study, this time carried out in *P. falciparum*, classified the gene as possibly dispensable^23^. To further our understanding of the biological function(s) of PfUT, we attempted to generate a conditional knock-down mutant by inserting the glucosamine-inducible glms ribozyme sequence in the 3′ untranslated region of *pfut*. The glms riboswitch has previously been used in *P. falciparum* to conditionally down-regulate the expression of several genes of interest^24–29^. Unexpectedly, insertion of the ribozyme sequence into the *pfut* gene locus was not inert, but instead resulted in 2.5-fold higher steady state transcript levels and associated with it 2.4-fold increased protein amounts, compared with the parental strain. We show that overexpression of *pfut* affected the length of the asexual intraerythrocytic life cycle by prolonging the S/M phase. In addition, merozoite invasion efficiency was reduced. Our data suggest that PfUT partakes in the regulatory network that controls merozoite invasion and cell cycle progression during schizogony.

## Results

### Generation of a conditional *pfut* knock-down mutant in *P. falciparum*

**Figure 1:**
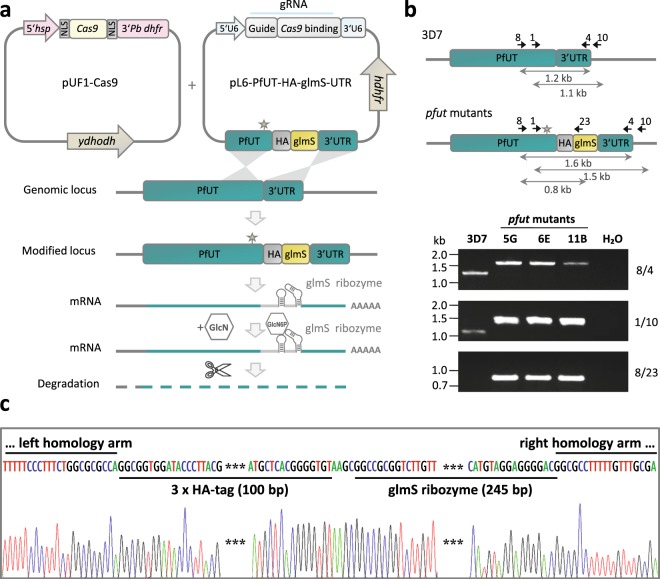
Generation of a conditional *pfut* knock-down mutant in *P. falciparum*. (**a**) Schematic representation of the CRISPR-Cas9-based strategy used to incorporate the HA tag and the glmS ribozyme sequences in the 3’ untranslated region of the endogenous *pfut* gene. The cloning strategy and the vectors used are described in the Materials and Methods section. The star indicates a shield mutation that prevents cleavage of the mutated *pfut* locus by Cas9. Glucosamine (GlcN) added to the culture medium is taken up by the parasite and converted to the glucosamine-6-phosphate (GlcN6P). Binding of GlcN6P stimulates self-cleavage of the glmS ribozyme, leading to mRNA destabilization and degradation of the transcript and associated with it, down-regulation of the corresponding protein. The GlcN dose-dependent growth curves performed to evaluate the optimal treatment conditions are shown in Supplementary Fig. 4. (**b**) Analysis of *pfut* mutants. The wild type and the genetically altered *pfut* locus are shown. The positions of relevant primers for analysis are indicated, as are the sizes of important PCR products. The integration event was verified by PCR, using genomic DNA from the resulting mutants, termed 5 G, 6E, 11B, and the parental 3D7 strain. The primer pairs used (8/4, 1/10 and 8/23) are indicated (see Supplementary Table 1 for more details on primers). Primers 8 and 10 are located upstream and downstream of the homology regions, respectively. Size markers are indicated in kilo base pairs (kb). (**c**) Representative DNA sequence chromatogram of one of the *pfut* mutants, showing the correct integration of the triple HA and the glmS ribozyme sequence into the 3′ untranslated region of *pfut*. This figure was reproduced from the PhD thesis by Jankowska-Döllken^32^.

In an effort to elucidate the biological function of PfUT, we generated a conditional knock-down mutant in the *P. falciparum* line 3D7, by inserting a triple hemagglutinin (HA) tag followed by the glmS ribozyme sequence in the 3′ untranslated region of *pfut*^27,30^, via CRISPR-Cas9 genome editing technology^31^ (Fig. 1a). Three independent clones, termed 5 G, 6E and 11B, were isolated by limiting dilution and the desired insertion of the glms ribozyme sequence was confirmed by sequence analysis of the genomic *pfut* gene locus^32^ (Fig. 1b,c). This approach followed six unsuccessful attempts each to generate *pfut* gene disruption or null mutants, using the selection-linked integration mediated targeted gene disruption (SLI-TGD) method^33^ or the CRISPR-Cas9 method to substitute serine for a functional Cys-3860 in the catalytic domain.

### *pfut* is overexpressed in the mutant lines

**Figure 2:**
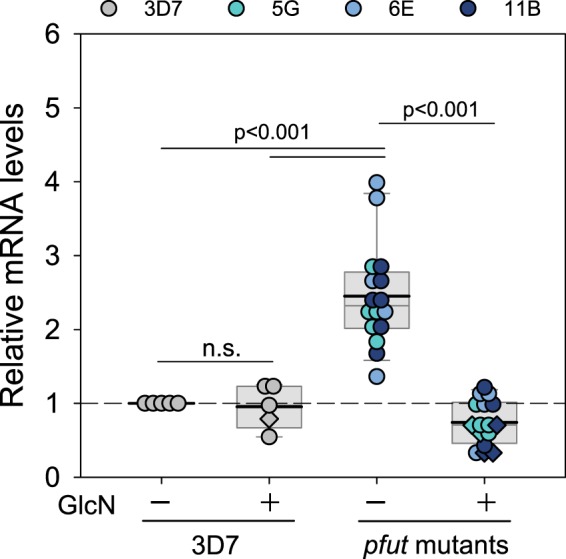
RT-qPCR analysis of steady state *pfut* transcript levels in the *pfut* mutant lines and the parental 3D7 strain. Total mRNA was isolated from the *pfut* mutants and 3D7 cultured in the presence or absence of 5 mM GlcN. The amount of steady state *pfut* transcripts were then quantified by RT-qPCR and normalized to the housekeeping gene β-tubulin followed by normalization to the *pfut* steady state RNA level in untreated 3D7. Trophozoites were analyzed throughout. Validation of the qPCR primers is depicted in Supplementary Fig. 6. Data derived from cells treated for 48 h (one cell cycle) and 120 h (three cell cycles) are indicated by rhombuses and circles, respectively. Each symbol represents an independent biological replicate. A box plot analysis is overlaid over the individual data points, with the median (thin grey line), mean (thick black line) and the 25% and 75% quartile ranges being shown. Statistical significance between the data sets was calculated using Holm-Sidak one way ANOVA. n.s. – not significant. This figure was reproduced from the PhD thesis by Jankowska-Döllken^32^.

We next assessed the potential of the inserted glms ribozyme sequence to down-regulate the expression of *pfut*. Steady-state RNA levels, indeed, fell by a factor of 3.3 to 30% of the initial value after either two or 5 days of incubation in the presence of 5 mM glucosamine (GlcN) (p < 0.001 according to one way ANOVA Holm-Sidak test), as determined by RT-qPCR (Fig. 2). However, the initial steady state RNA level in the absence of the inducer was 2.5-fold higher than that of the parental 3D7 line, with GlcN treatment having no effect on *pfut* mRNA levels in 3D7. Apparently, insertion of the glms ribozyme sequence in the 3′ untranslated region altered *pfut* RNA stability and/or transcription activity such that it resulted in an overexpression of the genetically altered gene.

**Figure 3:**
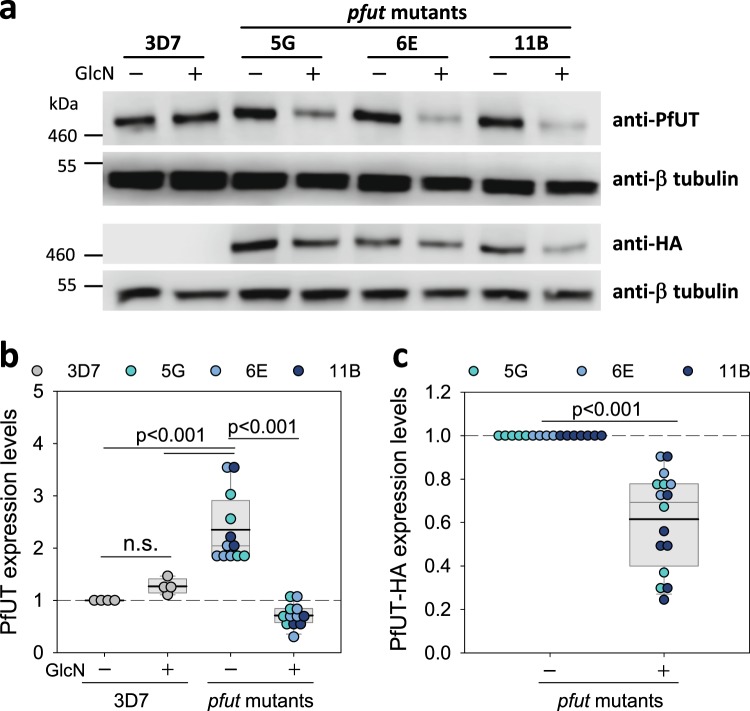
Steady state PfUT protein levels in the *pfut* mutants and the parental 3D7 strain. (**a**) Western blot analysis. Total protein lysates were prepared from late trophozoites cultured for 120 h in the presence or absence of 5 mM GlcN. Proteins were fractionated by SDS-PAGE and transferred to a PVDF membrane. The following antisera were used: anti-PfUT (raised against residues 473 to 712 of the N-terminal domain; rabbit; 1:1000); anti-α-tubulin (mouse, 1:1000); and anti-HA-tag (mouse, 1:1000). Representative Western blots are shown. Blots were cropped at the top and bottom to show the bands of interest. The uncropped, full-length blots are presented in Supplementary Fig. 9a (first and second panel) and 9b (third and fourth panel). A size marker is indicated in kDa. (**b**) The amount of steady state PfUT was quantified, on the basis of the anti-PfUT hybridization signal intensities, and normalized to the α-tubulin followed by normalization to steady state PfUT protein levels in untreated 3D7. Each symbol represents an independent biological replicate. A box plot analysis is overlaid over the individual data points, with the median (thin grey line), mean (thick black line) and the 25% and 75% quartile ranges being shown. Statistical significance between the data sets was calculated using Holm-Sidak one way ANOVA. n.s. – not significant. (**c**) As in (**b**) with the exception that the anti-HA signal intensities were analyzed and normalized to the corresponding α-tubulin signal followed by normalization to steady state PfUT-HA protein levels in untreated *pfut* mutants. This figure was reproduced from the PhD thesis by Jankowska-Döllken^32^.

Semi-quantitative Western blot analyses, using antibodies specific for PfUT and the internal control α-tubulin, confirmed overexpression of the genetically altered *pfut* gene in the mutant clonal lines (Fig. 3a,b). Compared to the parental strain 3D7, steady state PfUT protein levels were increased 2.4-fold in the mutants. The addition of GlcN decreased PfUT amounts 3.3-fold (by 70%) to a level slightly below the basal value observed in 3D7. Immunoblotting with antibodies against the HA-tag (expressed as a fusion protein with PfUT in the mutant lines) and α-tubulin corroborated the finding of GlcN down-regulating PfUT protein levels in the mutants (Fig. 3a,c). As an additional negative control, we investigated the influence of *pfut* overexpression and GlcN treatment on steady state protein levels of the chloroquine resistance transporter PfCRT, but observed no significant effects neither in 3D7 nor in the *pfut* mutants (Supplementary Fig. 2).

### PfUT is localized at the ER/Golgi complex in the mutants

**Figure 4:**
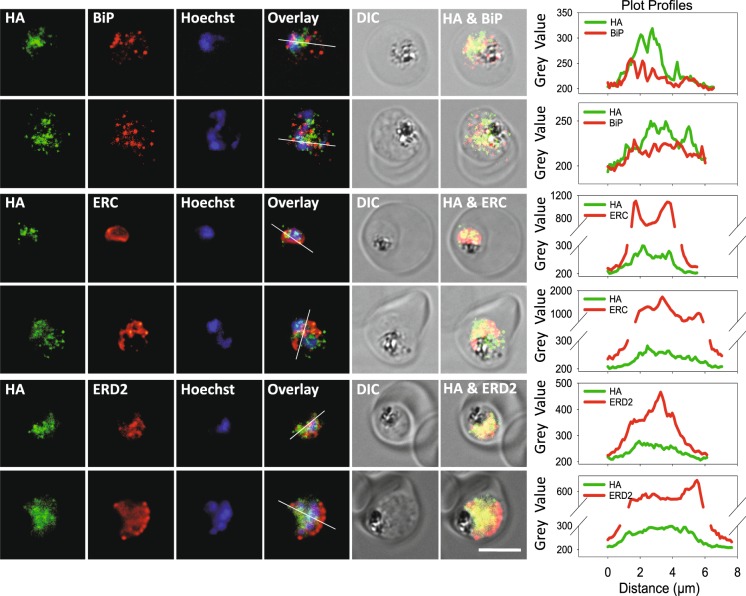
Colocalization of PfUT-HA with ER and Golgi markers in *pfut* mutants. Indirect immunofluorescence assay (IFA), using *pfut* mutants (at the trophozoite stage) and an anti-HA antiserum (mouse, 1:1000) together with the ER marker BiP (rabbit, 1:1000) or ERC (rabbit, 1:500), or with the Golgi marker ERD2 (rabbit, 1:500). Secondary antibodies were an anti-mouse Alexa Fluor 488 (green) and an anti-rabbit Alexa Fluor 546 (red). The nuclei were visualized with Hoechst (blue). Different channels, the differential interference contrast (DIC) image and two overlay images are shown for two representative examples each. The plot profiles are included for a better presentation of signal colocalization. Colocalization of anti-HA and anti-PfUT fluorescence signals is shown in Supplementary Fig. 7. Scale bar: 5 µm. This figure was reproduced from the PhD thesis by Jankowska-Döllken^32^.

**Figure 5:**
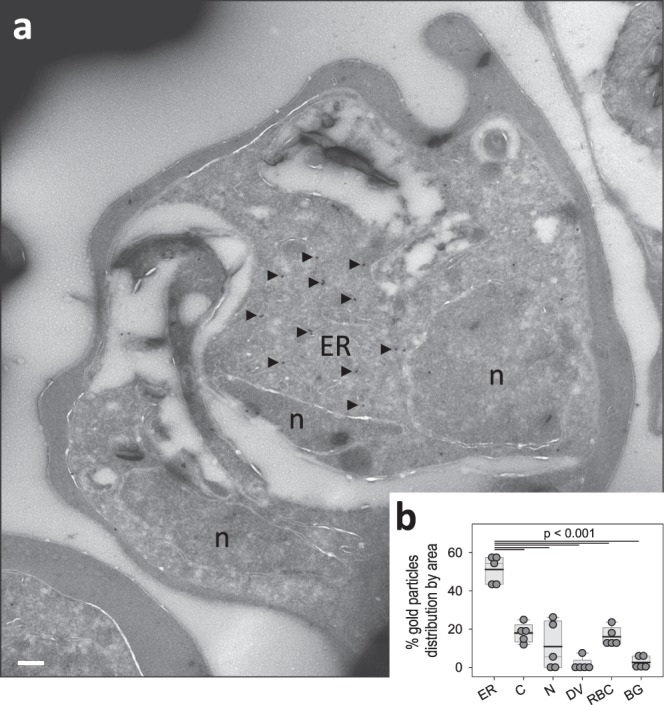
Subcellular localization of PfUT by immunoelectron microscopy. (**a**) A representative micrograph is shown of an erythrocyte infected with a *pfut* mutant. The sample was prepared according to the Tokuyasu protocol, and immunolabelled with anti-HA antiserum (mouse, 1:1), followed by staining with an anti-mouse antibody coupled to 10 nm colloidal gold (goat, 1:20). Arrowheads point towards gold label. n, nucleus; ER, endoplasmic reticulum/Golgi complex. Scale bar, 200 nm. (**b**) Quantification of immuno EM results. The distribution of gold grains was determined in 5 micrographs and analyzed according to their subcellular localization. Gold grains were significantly more present in areas of the ER/Golgi complex (ER) than in other subcellular compartments, including the parasite’s cytoplasm (C), nucleus (N), digestive vacuole (DV), red blood cell cytosol (RBC) and non-cellular background (BG). Each symbol represents data derived from an individual parasitized erythrocyte. A box plot analysis is overlaid over the individual data points, with the median (thin grey line), mean (thick black line) and the 25% and 75% quartile ranges being shown. Statistical significance between the data sets was assessed using the Holm-Sidak one way ANOVA test. This figure was reproduced from the PhD thesis by Jankowska-Döllken^32^.

A previous study has localized PfUT at the parasite’s ER/Golgi complex^15^. Immunofluorescence assays, using the conditional knock-down mutants, revealed comparable results, with the HA-tagged PfUT partially co-localizing with the ER markers, BiP and ERC, and the Golgi marker, ERD2 (Fig. 4), consistent with previous reports^15^. Immuno-electron microscopy using anti-HA antiserum confirmed a subcellular localization of PfUT at the ER/Golgi complex in the mutant lines (Fig. 5). A comparison of the micrographs with reference electron microscopic images of trophozoites^15,34,35^ provided no evidence of morphological abnormalities in the mutant parasite lines (Fig. 5), suggesting normal phenotypic characteristics of organelles and other subcellular compartments.

### *pfut* mutants displayed a slow proliferation phenotype

**Figure 6:**
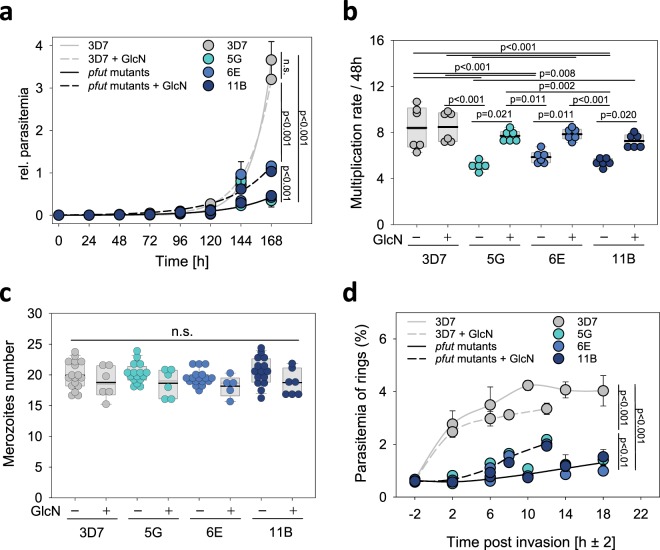
Phenotypic characterization of *P. falciparum pfut* mutants. (**a**) Asexual intraerythrocytic proliferation. Proliferation of the three independent *pfut* mutants (5 G, 6E and 11B) and the parental line 3D7 was determined over 168 h in the presence and absence of 5 mM GlcN. Time point 0 indicates the time point at which the cell culture was split and one aliquot was incubated in the presence of GlcN. The other aliquot served as the untreated control. The relative parasitemia represents the sum of the parasitemia in the culture, taking into account the splitting/dilution factors. Each symbol represents the mean ± SD of three independent biological replicates. A single two parameter exponential growth function was fitted to the data points. Statistical significance was assessed using the F-test. (**b**) Multiplication rate. The parasite multiplication rate (PMR) was determined for each clone as the fold increase in parasitemia per 48 h, measured over 4 cycles. (**c**) Merozoites number. The number of merozoites generated per schizont was determined in the presence and absence of 5 mM GlcN, using flow cytometry. Each symbol represents an independent biological replicate. A box plot analysis is overlaid over the individual data points, with the median (thin grey line), mean (thick black line) and the 25% and 75% quartile ranges being shown. Statistical significance was assessed using Holm-Sidak one way ANOVA. n.s. – not significant. (**d**) Merozoite invasion efficiency. Synchronized parasites at the late schizont stage were allowed to re-invade and the number of ring stage parasites was quantified over time. The mean ± SD of three independent biological replicates is shown. Statistical significance was assessed using the F-test. This figure was reproduced from the PhD thesis by Jankowska-Döllken^32^.

We next investigated the effect of *pfut* overexpression on parasite development. Interestingly, the *pfut* mutants grew significantly slower in the absence of GlcN, compared with the parental 3D7 line (p < 0.001 according to F-test statistics) (Fig. 6a and Supplementary Fig. 3). The slow proliferation phenotype was partially reversed in the presence of GlcN (5 mM) (Fig. 6a). Proliferation of the 3D7 was unaffected by GlcN treatment (Fig. 6a and Supplementary Fig. 4).

To better understand the underlying basis of the proliferation defect displayed by the *pfut* mutants, we determined the parasite multiplication rates and the merozoite numbers generated per schizont^36–38^. The parental 3D7 line multiplied with a rate of 8 ± 1, regardless of the presence of GlcN, consistent with previous reports^37^. In comparison, *pfut* mutants displayed significantly lower parasite multiplication rates of 5 ± 1 (p < 0.001 according to one way ANOVA Holm-Sidak test) and only in the presence of GlcN did their multiplication rates approach values comparable to that of 3D7 (Fig. 6b). In contrast to the multiplication rates, the average number of merozoites generated during schizogony did not differ between *pfut* mutants and 3D7 (Fig. 6c). In all cases merozoite numbers of 19 ± 2 were observed, consistent with previous determinations for 3D7^38,39^. However, the merozoite invasion efficiency was low in the *pfut* mutants and, while it increased in the presence of GlcN, it remained below that of the 3D7 control (Fig. 6d), as determined in synchronized cultures by quantifying the number of ring stages in the next cycle. Since multiple infections could falsify the invasion rate, we quantified the proportion of single, double and triple infections, but found comparable values for both the *pfut* mutants and the parental line 3D7 (Supplementary Fig. 5).

### Slow proliferation is associated with a prolonged S/M-phase in *pfut* mutants

**Figure 7:**
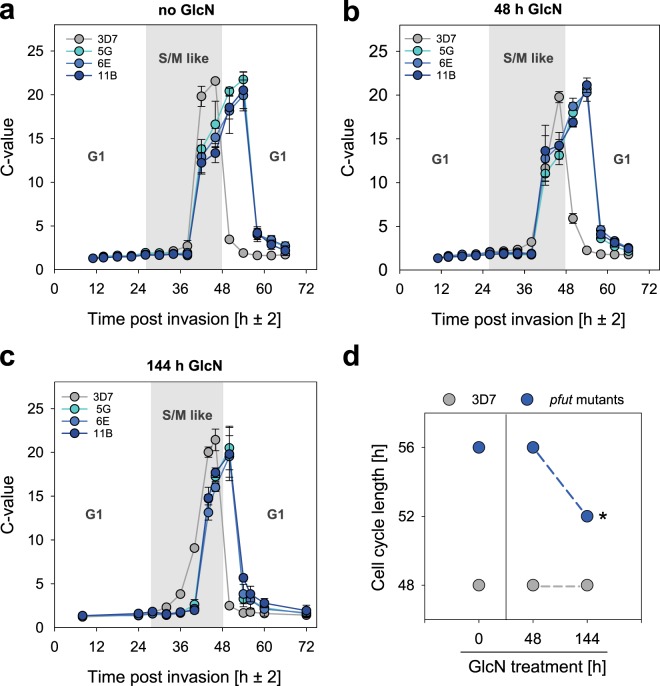
Analysis of the cell cycle length in *pfut* mutants and 3D7. The DNA copy number (C-value) was determined in highly synchronized cells in intervals of 4 h throughout the 48 h replicative cycle. (**a**) Cell cycle in the absence of GlcN; (**b**) after one cycle in the presence of 5 mM GlcN; (**c**) after three cycles in the presence of 5 mM GlcN. The drop in the C-value after reaching the peak indicates the beginning of a new cycle. Each data point represents the mean ± SD of three independent biological replicates. The G1 and S/M-like phase of the 48 h intraerythrocytic cell cycle of 3D7 are indicated to better display the differences with *pfut* mutants. (**d**) Cell cycle length as a function of GlcN treatment for 48 h (one replicative cycle) and 144 h (three replicative cycles) in *pfut* mutants and 3D7. ^*^p < 0.001, compared with the untreated *pfut* mutant cell lines, according to Student’s t-test. This figure was reproduced from the PhD thesis by Jankowska-Döllken^32^.

We next investigated whether the slow proliferation phenotype displayed by *pfut* mutants can also be attributed to changes in cell cycle duration. To this end, we measured the DNA content of the parasite in highly synchronized cultures in intervals of 4 h throughout the 48 h replicative cycle, using flow cytometry. The resulting DNA copy number value or C-value was then analysed as a function of time post invasion. In 3D7, the C-value increased from 1 to 2 around 26 ± 2 h post invasion (Fig. 7a) as DNA replication commenced with the beginning of the S/M-like phase, consistent with previous reports^40,41^. The C-value then peaked at 46 ± 2 h post invasion before it rapidly dropped^21,42^ (Fig. 7a), which marks the end of the replicative cycle and the rupture of the infected erythrocyte and the release of daughter merozoites. In the case of the *pfut* mutants, the replicative cycle was extended by approximately 8 h and instead of 48 h lasted 56 h (Fig. 7a,d). The extended intraerythrocytic life cycle was associated with a prolonged S/M-phase and a slower overall DNA replication rate, whereby the S/M-phase commenced at the same time point post invasion and C-values reached comparable peaks, in both 3D7 and *pfut* mutants (Fig. 7a and Table 1). In parallel experiments, we investigated the cell cycle characteristics in cells treated with GlcN for one or three replicative cycles (Fig. 7 and Supplementary Fig. 3). No differences were observed after treatment for one cycle, with the S/M phase still being prolonged by approximately 8 h in the mutants, compared with 3D7 (Fig. 7b and Table 1). In comparison, exposure to GlcN for three cycles partially restored the length of the S/M phase and the cell cycle to 24 h and 52 h, respectively, in *pfut* mutants (Fig. 7c,d and Table 1). The cell cycle length remained unaffected by GlcN treatment in 3D7.

**Table 1:** Cell cycle characteristics for *pfut* mutants and 3D7.

Treatment	Clone	Onset of DNA replication [h p.i.]	Peak of the C-value [h p.i.]	Length of the S/M-like phase [h]
1st cycle (−GlcN)	3D7	26 ± 2	46 ± 2	20 ± 2
	5 G	26 ± 2	54 ± 2*	28 ± 2*
	6E	26 ± 2	54 ± 2*	28 ± 2*
	11B	26 ± 2	54 ± 2*	28 ± 2*
1st cycle (+GlcN)	3D7	26 ± 2	46 ± 2	20 ± 2
	5 G	26 ± 2	54 ± 2*	28 ± 2*
	6E	26 ± 2	54 ± 2*	28 ± 2*
	11B	26 ± 2	54 ± 2*	28 ± 2*
3rd cycle (+GlcN)	3D7	28 ± 2	46 ± 2	18 ± 2
	5 G	28 ± 2	50 ± 2*^,#^	22 ± 2*^,#^
	6E	28 ± 2	50 ± 2*^,#^	22 ± 2*^,#^
	11B	28 ± 2	50 ± 2*^,#^	22 ± 2*^,#^

The following parameters are indicated: the onset of DNA replication, as defined by the time point post invasion (p.i.) when the C-value reaches 2; the time point of maximal C-value; and the length of the S/M phase. Data are shown for cells cultured in the absence of GlcN and cells treated for one and three replicative cycles with 5 mM GlcN. ^*^p < 0.001, compared with the parental line 3D7, according to Holm-Sidak one way ANOVA. ^#^p < 0.001, compared with untreated *pfut* mutant cultures, according to Holm-Sidak one way ANOVA.

## Discussion

There have been conflicting results of whether or not *pfut* is essential for intraerythrocytic development of *P. falciparum*. Whereas studies conducted in *P. berghei* have suggested a critical function for the PfUT homologue in the biology of the mouse malaria parasite^22^, studies performed in *P. falciparum* have concluded that PfUT is likely dispensable during intraerythroyctic development, on the basis of the accessibility of the corresponding gene locus to *piggyBac* transposon mutagenesis^23^ (Supplementary Fig. 1), although it is not clear whether the latter strategy resulted in null mutants or partially active mutants that still maintained some PfUT activity. To assess the importance of PfUT during blood stage development, we applied the SLI-TGD method to the *pfut* locus in 6 independent experiments, but did not obtain the desired disruption mutation, which according to criteria defined by Birnbaum *et al*.^33^ suggests that *pfut* is an essential gene during intraerythrocytic development. Further suggesting an essential role of *pfut* during blood stage development, the active, thioester intermediate forming amino acid cysteine at position 3860 within the catalytic HECT domain could not be replaced with serine, in spite of three attempts and the fact that the same CRISPR-Cas9 guide RNA was used as for the successful integration of the glms ribozyme sequence in the 3′ end of the *pfut* locus.

The intention to integrate the glms ribozyme in the 3′ end of the *pfut* locus was to generate a conditional knock-down mutant for further functional analyses^32^ (Fig. 1). While we got the mutant and although treatment of the cells with glucosamine down-regulated *pfut* expression levels by a factor of 3.3, a knock-down was not obtained in stricto sensu as integration of the glms ribozyme sequence led to approximately 2.5-fold increased steady state RNA and protein levels, compared with the parental 3D7 strain (Figs. 2 and 3a,b). It seems that the genetic manipulation stabilized the mRNA and protected it from degradation or, alternatively, stimulated transcriptional activity. Other studies have also noted an effect of the glms ribozyme on expression levels, at least in qualitative terms^28,29^.

Altering the gene locus did not affect trafficking or sub-cellular localization of PfUT, with PfUT being localized at the ER/Golgi complex (Figs. 4 and 5), consistent with previous reports^15^.

Which essential role *pfut* plays during parasite development is still unclear. Previous studies have ascribed PfUT a function in the ubiquitin fusion degradation pathway, on the basis of homologies to the characterized HECT E3 ubiquitin-protein ligase UFD4 of *S. cerevisiae*^16^ and because PfUT interacts with components of the proteasome in a yeast-two-hybrid screen^43^. Another study has implicated PfUT, respectively polymorphisms within PfUT, in reduced responsiveness to quinine and its enantiomer quinidine^15^. Our finding that the expression level of *pfut* can modulate the cell cycle length by prolonging the S/M-like phase was unexpected (Fig. 7). However, this finding is consistent with the reported role of E3 ubiquitin ligases, including those of the HECT subclass, in signalling pathways that regulate cell cycle progression, as shown in other system^44–47^. In particular the G1/S transition is tightly controlled by the ubiquitin proteasome system^48^. However, the onset of DNA replication was not altered in the *pfut* mutants (Fig. 7 and Table 1), suggesting a functional, non-PfUT dependent checkpoint between the G1 and S/M phase. Thus, PfUT seems to participate in the network that coordinates schizogony, the unconventional cell division by which *P. falciparum* and other malaria parasites reproduce and which is characterized by multiple rounds of DNA replication and nuclear division without cytokinesis.

Previous studies have identified several factors that control cell cycle progress in *P. falciparum*. This includes cyclins (encoded by *pfcyc1-4* genes), cyclin-dependent protein kinases (CDKs), CDK-related kinases (CRKs)^40,41,49–53^, calcium-dependent protein kinases (CDPKs)^54,55^ or the cyclic GMP-dependent protein kinase PfPKG^41,56^. Which, if any, of these factors are targeted by PfUT for degradation remains to be seen. In this context it is noteworthy that *in silico* studies have predicted an interaction between PfUT and a plasmodial homologue of cell division cycle 48 (CDC48)^7^. As shown in other systems, CDC48, an AAA-type ATPase, is involved in the ERAD pathway by pumping misfolded and polyubiquitinated proteins from the ER into the cytoplasm where these proteins are degraded by the proteasome^7,8,57,58^. Notably, in yeasts, CDC48 has been associated with the degradation of cell cycle regulators^59^.

We further noted an effect of *pfut* on merozoite invasion efficiency (Fig. 6d). Interestingly, a yeast two-hybrid screen has previously identified the merozoite surface protein 1 (MSP1) and the rhoptry neck protein RON2 as interaction partners of PfUT^43^. Both proteins are critically involved in merozoite egress and/or invasion^60–64^. For instance, MSP1 destabilizes the membrane skeleton of the host erythrocytes, which, in turn, promotes red blood cell rupture and merozoite release^60^. Other studies have shown that MSP1 recruits variable peripheral proteins and that the ensuing supermolecular complex interacts with ligands on the red blood cell, in particular glycophorin A and band 3, to initiate the invasion process by the merozoite^61,62,65^. RON2 forms a complex with the apical membrane antigen 1 (AMA1), which plays a pivotal role during invasion by triggering junction formation^63^. In summary, our data suggest a role of PfUT in merozoite invasion and cell cycle coordination. Overexpression of PfUT distorts these processes, possibly, by PfUT targeting, accidentally or in an untimely fashion, factors critical for invasion and cell cycle regulation for degradation, thereby prolonging the S/M phase and reducing merozoite invasion efficiency.

## Methods

### *In vitro* culture of *P. falciparum*

Parasites were cultured in human A + erythrocytes and complete RPMI medium (10% human A serum, 0.2 μg ml^−1^ gentamycin, 0.1 mM hypoxanthine) at 2–4% haematocrit, according to standard protocols^66^. Human red blood cells and human sera were purchased from the Deutsche Rote Kreuz-Blutspendedienst Baden-Württemberg/Hessen GmbH. Parasite cultures were synchronized using 5% D-sorbitol^67^. For tight synchronization, a combination of sorbitol and heparin (50 U ml^−1^) was used^68,69^.

### Oligonucleotides

Primers used for PCR amplification, sequencing and qPCR are depicted in Supplementary Table 1.

### Generation of the glms ribozyme transfection construct

The appropriate homology regions were amplified from genomic DNA of 3D7, using primers 1/2 and 3/4 and cloned into the pL6-HA-glmS plasmid, using restriction endonucleases SpeI/BssHII and NarI/AflII, respectively. The guide RNA (primers 5/6) was cloned into the BtgZI linearized final pL6-PfUT-HA-glmS-UTR vector, using the *In Fusion* cloning technology (Clontech Laboratories).

### Transfection of *P. falciparum*

*P. falciparum* ring stage parasites at 3–5% parasitemia were electroporated with 100 µg of the appropriate transfection vectors (pL6 and pUF1-Cas9), as previously described^70^. Transfectants were selected using 5 nM WR99210 and 1.5 μM DSM1^31^ and maintained in RPMI medium supplemented with 5% human A serum, 0.25% Albumax I, 0.2 μg ml^−1^ gentamycin and 0.2 mM hypoxanthine. Clonal parasite populations were obtained by limiting dilution. The genetically engineered *pfut* locus was confirmed for each clone by sequencing analysis, using primers 8–10 and 22–25, after amplification using total genomic DNA and primers 8 and 10.

### RT-qPCR

Total RNA was extracted from trophozoite-stage *P. falciparum* cultures, using the Trizol-chloroform method. The cells were centrifuged at 4 °C for 2 min at 900 × g, with brake-off at 130 × g. The pellet was lysed in ice cold 0.2% saponin, washed in ice cold PBS and finally resuspended in the Trizol reagent. For phase separation, 200 µl of chloroform were added per 1 ml of Trizol. The mixture was vigorously shaken by hand, incubated for 3 min at RT and centrifuged at 10500 × g for 30 min at 4 °C. The upper aqueous phase containing RNA was collected and precipitated by adding 500 µl of isopropanol per 1 ml of Trizol. The mixture was incubated at −80 °C for at least 30 min and subsequently centrifuged at 10500 × g for 10 min at 4 °C. The pellet was washed with cold 70% ethanol and centrifuged at 10500 × g for 10 min at 4 °C. The RNA pellet was then air-dried at RT to eliminate any trace of ethanol, and dissolved in 30 µl of ribonuclease-free water. The possible genomic DNA contamination was removed from the freshly isolated RNA samples using the DNase Treatment and Removal Kit (Invitrogen), according to the manufacturer’s instructions. Subsequently, the cDNA was synthesized using the SuperScript III First-Strand Synthesis SuperMix (Invitrogen), following the protocol provided by the manufacturer. The cDNA concentration was measured by spectrophotometry and adjusted to 50 ng µl^−1^. A quantitative real-time PCR was performed using the ABI 7500 Real-Time PCR detection system and the FastStart Universal SYBR Green Master ROX (Roche). Samples were analyzed in 96-well plates, in technical triplicates (each containing 200 ng of cDNA), using the following primer pairs: for *pfut*, primers 26/27; for *β-tubulin*, primers 28/29. A control without template DNA was analysed in parallel as a negative control. The qPCR thermocycling conditions included polymerase activation at 95 °C for 10 min, followed by 40 cycles of denaturation (95 °C for 15 sec), annealing (55 °C for 15 sec) and elongation (60 °C for 45 sec). Data were analyzed using the delta-delta Ct method taking into account primers efficiencies (Supplementary Fig. 6).

### Western blot

*P. falciparum* trophozoite stage parasites were purified using the magnetic activated cell sorting (MACS) system (Miltenyi Biotec). Parasitized erythrocytes were lysed with 0.7% saponin in the presence of protease inhibitors (Roche) for 3 min on ice, centrifuged for 1 min at 17000 × g at 4 °C and washed 3 times in ice cold PBS containing protease inhibitors. Parasites were then lysed in 4 pellet volumes of RIPA buffer (50 mM Tris pH 7.5, 150 mM NaCl, 5 mM EDTA, 50 mM NaF, 0.5% NaDOC, 0.1% SDS, 1% Triton X-100, 2 mM DTT, 1 mM PMSF, protease inhibitors) containing 10 µg ml^−1^ DNase I for 30 min on ice and centrifuged for 20 min at 17000 × g at 4 °C. Protein lysates (supernatant) were transferred to a fresh Eppendorf tube, resuspended in 1:1 ratio with 2x Protein Loading Buffer (3% SDS, 250 mM Tris pH 6.8, 20% Glycerol, 0.1% Bromophenol blue) containing β-mercaptoethanol and stored at −20 °C. Samples and the HiMark Pre-Stained HMW Protein Standard (31 to 460 kDa; Invitrogen) were loaded on NuPAGE Tris-Acetate 3–8% gradient gels (Invitrogen). Gels were run at 150 V and 40 mA for approx. 1.5 h. Protein transfer from the gel onto a PVDF membrane was carried out for 1 h at 230 mA and 30 V using XCell II Blot Module (Thermo Fisher Scientific). The membrane was blocked in 5% milk and incubated with the respective primary and subsequently secondary antibodies diluted in 1% BSA. The following antibodies were used: mouse anti-α-tubulin (1:1000; Sigma Aldrich), mouse anti-HA clone 12CA5 (1:1000; Roche), rabbit anti-PfUT clone 1–2a raised against residues 473 to 712 of the N-terminal domain (1:1000)^15^, guinea pig anti-PfCRT (1:1000; custom-made by Eurogentec)^71^, rabbit anti-Ub FL-76 (1:2000; Santa Cruz Biotechnology), goat anti-mouse-POD (1:10000; Jackson ImmunoResearch), goat anti-rabbit-POD (1:10000; Jackson ImmunoResearch) and donkey anti-guinea pig-POD (1:5000; Jackson ImmunoResearch). The mouse anti-HA and the rabbit anti-PfUT antibodies colocalized at PfUT (Supplementary Fig. 7). Signals were detected using the LiCor C-DiGit Blot Scanner and images were processed using Image Studio software.

### Immunofluorecence assay (IFA)

Parasites at the trophozoite stage were either purified using the MACS system or collected unpurified from erythrocytes at a parasitemia of 5–10%. Samples were centrifuged at 900 × g for 2 min. Erythrocytes were washed in PBS, followed by fixation in 4% paraformaldehyde and 0.0075% glutaraldehyde for 30 min at RT, with rotation. Samples were then washed twice with PBS and kept at 4 °C or directly incubated with 0.1% Triton X-100 and 125 mM glycine for 15 min at RT, with rotation. All the following treatments were conducted at RT, with rotation. 3% BSA in PBS was used as blocking buffer and as antibodies dilution buffer. Samples were blocked for at least 2 h, followed by primary antibody staining for 1.5 h (or at 4 °C overnight). Cells were then washed in 3% BSA in PBS 3 times for 15 min, followed by secondary antibody staining for 45 min in the dark. Samples were then washed with PBS 3 times for 15 min, including addition of 5 µM Hoechst to the final wash. Cells were resuspended in PBS and stored at 4 °C until imaging. Samples were imaged using the Carl Zeiss Axiovert 25 widefield miscroscope using objective with 63x magnification. Images were acquired and processed in FIJI. The following antibodies were used: mouse anti-HA clone 12CA5 (1:1000; Roche), rabbit anti-PfUT N-terminal clone 1-2a (1:1000)^15^, rabbit anti-BiP (1:1000)^72^, rabbit anti-PfERC (1:500)^73^, rabbit anti-PfERD2 (1:500)^74^, goat Alexa Fluor 488 anti-mouse IgGs (1:1000; Invitrogen) and goat Alexa Fluor 546 anti-rabbit IgGs (1:500; Invitrogen).

### Transmission electron microscopy (TEM)

Immunogold-labelled samples for transmission electron microscopy (TEM) were prepared according to the Tokuyasu method^75^. To this end, erythrocytes infected with trophozoites were purified from uninfected red blood cells using MACS system. After elution from the column, parasitized erythrocytes were centrifuged for 2 min at 900 × g and the pellet was washed twice with 1x PHEM (60 mM PIPES, 25 mM HEPES, 10 mM EGTA and 2 mM MgCl_2_, pH 6.9) buffer. The sample was fixed in 4% paraformaldehyde and 0.016% glutaraldehyde in 1x PHEM buffer for 1 h at RT under agitation and subsequently washed twice in 1x PHEM. Pellet was resuspended in 1:1 ratio in 1x PHEM followed by embedding in 10–12% gelatine. Samples were incubated for 5 min at 37 °C, shortly pelleted and solidified on ice. Gelatinized samples were cut into small cubes (around 1 mm^3^) and infused with a 2.3 M sucrose solution for cryo-protection (at 4 °C with light agitation overnight). The cubes were mounted on metal pins with excess of sucrose solution and frozen in liquid nitrogen. Subsequently, the frozen cubes were sectioned, using the cryo-ultramicrotome (Leica, UCT6), melted on a drop of sucrose/methyl cellulose solution (2.3 M/1.5%) and mounted onto TEM grids coated with Formvar film. Immuno-labelling was preceded by incubation in quenching buffer (50 mM glycine in PBS) for 30 min to quench the remains of free aldehyde fixatives, followed by soaking in blocking buffer containing 1.5% BSA and 0.1% fish skin gelatine in 1x PBS for 30 min. This blocking buffer was also used as a dilution buffer for antibodies. Tokuyasu cryo-sections were labelled with primary mouse anti-HA antibody (dilution 1:1) for 1 h at RT and washed 5 times 3 min in the quenching buffer. Subsequent labelling with secondary goat anti-mouse antibody coupled to 10 nm colloidal gold (dilution 1:20) was carried out for 1 h at RT followed by washing 3 times in quenching buffer and 3 times in ddH_2_O, for 3 min each. Cryo-sections were then fixed in 1% glutaraldehyde for 5 min at RT, followed by washing 5 times for 3 min in ddH_2_O. To enhance the contrast and to embed the samples, the sections were washed in a mixture of uranyl acetate and methyl cellulose (1.8%/0.8%) and air dried. Specimens were examined and images recorded using Jeol JEM-1400 transmission electron microscope. ER/Golgi complex was determined as membranous compartments adjacent to nuclei, including the margin of 50 nm at each side of a membrane for the accuracy of labelling that takes into consideration the cumulative length of the primary and secondary antibodies and the size of the protein A gold marker. The distribution of gold particles was analyzed stereologically according to their subcellular localization in the infected red blood cell on several micrographs. For this purpose, a square grid with known spacing was placed over each of the micrographs to facilitate the quantification of gold hits and the number of line intersections which define the area of a compartment. For each compartment, gold counts were determined and depicted as percentage of the total number of gold particles in the analyzed area, as well as was the compartment area presented as percentage of the total analyzed area. Subsequently, the ratios of gold counts per compartment area were generated for each subcellular region to obtain the number of gold hits per unit area. These values were then depicted as a proportion of total gold signal measured across all analyzed compartments of several micrographs.

### Flow cytometry based analyses

Parasite DNA was stained using SYBR Green, as described by Ganter *et al*.^41^. Parasites were fixed in 4% paraformaldehyde and 0.0075% glutaraldehyde at 4 °C overnight. Samples were then washed several times with PBS and stored in PBS at 4 °C. 200 µl of each sample were transferred to a 96-well V-bottom plate, centrifuged for 2 min at 900 × g and permeabilized with 200 µl of 0.1% Triton X-100 for 8 min at RT. Cells were washed twice with PBS, and subsequently treated with 200 µl of 0.3 mg ml^−1^ RNase A for 30 min at 37 °C. Samples were then washed twice with PBS and kept at 4 °C until staining. Cells were incubated with 200 µl of SYBR Green I (1:2000 in PBS) for 20 min at RT in the dark. Stained samples were then washed twice with PBS, transferred to FACS tubes containing 1 ml of cold PBS and run on a BD FACSCanto flow cytometer. SYBR Green fluorescence was detected in the fluorescein isothiocyanate (FITC) channel. For each sample 50,000 events were recorded and analyzed using Flowing Software. The FSC-H versus FSC-A plot was first used to discriminate doublets from single cells. Singlets gate was further used for analysis of FITC positive events. The unstained sample served as a negative control used for gating of uninfected erythrocytes.

The parasitemia was quantified as a ratio of FITC positive cells representing infected erythrocytes to the total number of red blood cells analyzed per one flow cytometry measurement. In addition, we distinguished between single, double, and triple *P. falciparum* infections^76^. For the analysis of multiple infections, highly synchronized ring stage parasite cultures were used. The populations of single-, double- and triple-infected erythrocytes were determined via a defined gating on dot plot and histogram (Supplementary Fig. 8).

In order to quantify the parasite multiplication rates (PMR), parasite cultures were synchronized using sorbitol and heparin treatments and diluted to 0.1% parasitemia at the trophozoite stage. 200 µl of each of three independent biological replicates were collected every 48 h over 4 cycles, fixed, stained with SYBR Green and run on a BD FACSCanto flow cytometer. Data were analyzed using Flowing Software, Excel and Sigma Plot 13. Data points were plotted in a scatter plot depicting parasitemia expressed as the natural logarithm over the time of 4 replicative cycles. A linear regression was then fitted to the data points and the parasite multiplication rate (PMR) was quantified as *PMR* = *e*^*a*^, where *a* corresponds to the slope of the linear regression.

The number of merozoites generated per schizont was determined via flow cytometric analysis of the DNA content in mature schizonts. Parasites were synchronized using sorbitol and heparin treatments. Samples were collected, fixed, stained with SYBR Green and run on a BD FACSCanto flow cytometer. Data were analyzed using Flowing Software and Sigma Plot 13. The merozoites number was expressed as a mean fluorescence emitted by a population of schizonts (containing multiple DNA copy numbers), divided by a mean intensity obtained for a population of single-infected red blood cells (with DNA copy number of one). The populations of schizonts and single-infected erythrocytes were determined via a defined gating strategies (Supplementary Fig. 8).

Cell cycle progression of *P. falciparum* can be tracked using flow cytometry, by quantification of the increase in DNA content over time. In order to determine the intraerythroycytic cycle duration, parasite cultures were synchronized within a 4 h window, using sorbitol and heparin, and adjusted to 0.5% ring stage parasitemia. The cell cycle length was analyzed as previously described^41^, by measuring the parasite DNA content (C-value) at 4 h intervals over at least 60 h. At each time point, 200 µl of each of three independent biological replicates were collected, fixed, stained and run on a BD FACSCanto. Data were analyzed using Flowing Software, Excel and Sigma Plot 13. The C-value was quantified by dividing the total parasite mean fluorescence intensity at particular time point by the mean intensity of a single infection from the first time point. The visible drop of the C-value after reaching the peak at schizont stage indicates the beginning of a new cycle.

### Statistical analysis

Data are given as mean ± standard deviation (SD) throughout this study, if not indicated otherwise. The independent biological replicates are indicated as individual data points on the figures. Statistical analyses were performed, using the Sigma Plot (v.13, Systat) software. Statistical significance was assessed using the Holm–Sidak one way analysis of variance test, Student’s t-test or F-test, where indicated.

## Supplementary information

supplementary information

## Data Availability

The authors declare that the data supporting the findings of this study are available within the article and supplementary information files, or are available from the authors upon request.
