# Supplementary material for: Overexpression of the HECT ubiquitin ligase PfUT prolongs the intraerythrocytic cycle and reduces invasion efficiency of Plasmodium falciparum
Source: Sci Rep. 2019 Dec 4;9:18333. doi: 10.1038/s41598-019-54854-z (PMC6893019; doi:10.1038/s41598-019-54854-z)
Supplement: Supplementary file 1 — supplementary information [file 41598_2019_54854_MOESM1_ESM.docx]

**Overexpression of the HECT ubiquitin ligase PfUT prolongs the intraerythrocytic cycle and reduces invasion efficiency of *Plasmodium falciparum***

Monika Jankowska-Döllken, Cecilia P. Sanchez, Marek Cyrklaff and Michael Lanzer

**Supplementary Table 1. Primers used for cloning and analysis.**

*(blue: enzyme restriction sites; green: homology regions for In Fusion cloning; red: desired mutations)*

| **No.** | **Name** | **Sequence** |
| --- | --- | --- |
| 1 | UT-12580- SpeI-for | GGACTAGTTTAATGAAAGTTGTTAAGAAGGAG |
| 2 | UT-13040-BssHII-rev | TTGGCGCGCCAGAAAGGGAAAAATTCTTTTGGC |
| 3 | UT-3'UTR-NarI-for | ATGCGGCGCCTTTTTGTTTGCGACCCGATG |
| 4 | UT-3'UTR-AflII-rev | CAGTCTTAAGTCTTTAATGTATATGTCCTTGAC |
| 5 | UT-guide1-for | TAAGTATATAATATTAAGAAAGTGAAAAGTTCTTTGTTTTAGAGCTAGAA |
| 6 | UT-guide1-rev | TTCTAGCTCTAAAACAAAGAACTTTTCACTTTCTTAATATTATATACTTA |
| 7 | UT-guide1-rev-short | TAAAACAAAGAACTTTTCACTTTC |
| 8 | UT-12367-for | ATCTGCCCTACCTAATAATGG |
| 9 | UT-3’UTR-rev | AACATTTGGGGGAATCTCTC |
| 10 | UT-3'UTR-rev-2 | AAATACTCTTAGGAACTTAAACC |
| 11 | UT-12265-SpeI-for | ACTAGTTAGATATACGAACGATTCAATTAC |
| 12 | UT-12614-C2S-for | TACCAAGTGTGATGACTTCTAC |
| 13 | UT-12614-C2S-rev | GTAGAAGTCATCACACTTGGTA |
| 14 | UT-61-NotI-for | GCGGCCGCTAAAATGGAGATGAATCTGAAATGG |
| 15 | UT-498-MluI-rev | ACGCGTCTACCTACAACCTCATTTATCC |
| 16 | UT-548-rev | GCATAAACTCCCATATGCTCTTG |
| 17 | UT-5’UTR-for | TCTTTATTTCTACATCATGCTTTAAG |
| 18 | pJET-for | CGACTCACTATAGGGAGAGCGGC |
| 19 | pJET-rev | AAGAACATCGATTTTCCATGGCAG |
| 20 | pL6-guide-for | gtaaccaaaatgcataatttttcc |
| 21 | pL6-guide-rev | taggaaataataaaaaagcacc |
| 22 | pL6- HA-glmS-5’-for | atttaactatatactatggaatac |
| 23 | pL6- HA-glmS-5’rev | tattgagaaaataagaacaagac |
| 24 | pL6-HA-glmS-3’-for | atcacatgatcttccaaaaaacatg |
| 25 | pL6-HA-glmS-3’-rev | taaaccaatagataaaatttgtagag |
|  |  |  |
|  |  |  |

**Primers used for qPCR:**

| **No.** | **Name** | **Sequence** |
| --- | --- | --- |
| 26 | UT-10-for | TACTTGCTTTTTGAGAATTCCCAG |
| 27 | UT-309-rev | ATTGCTCAAACCTCCGTCAG |
| 28 | β-tubulin-for | TGATGTGCGCAAGTGATCC |
| 29 | β-tubulin-rev | TCCTTTGTGGACATTCTTCCTC |
|  |  |  |


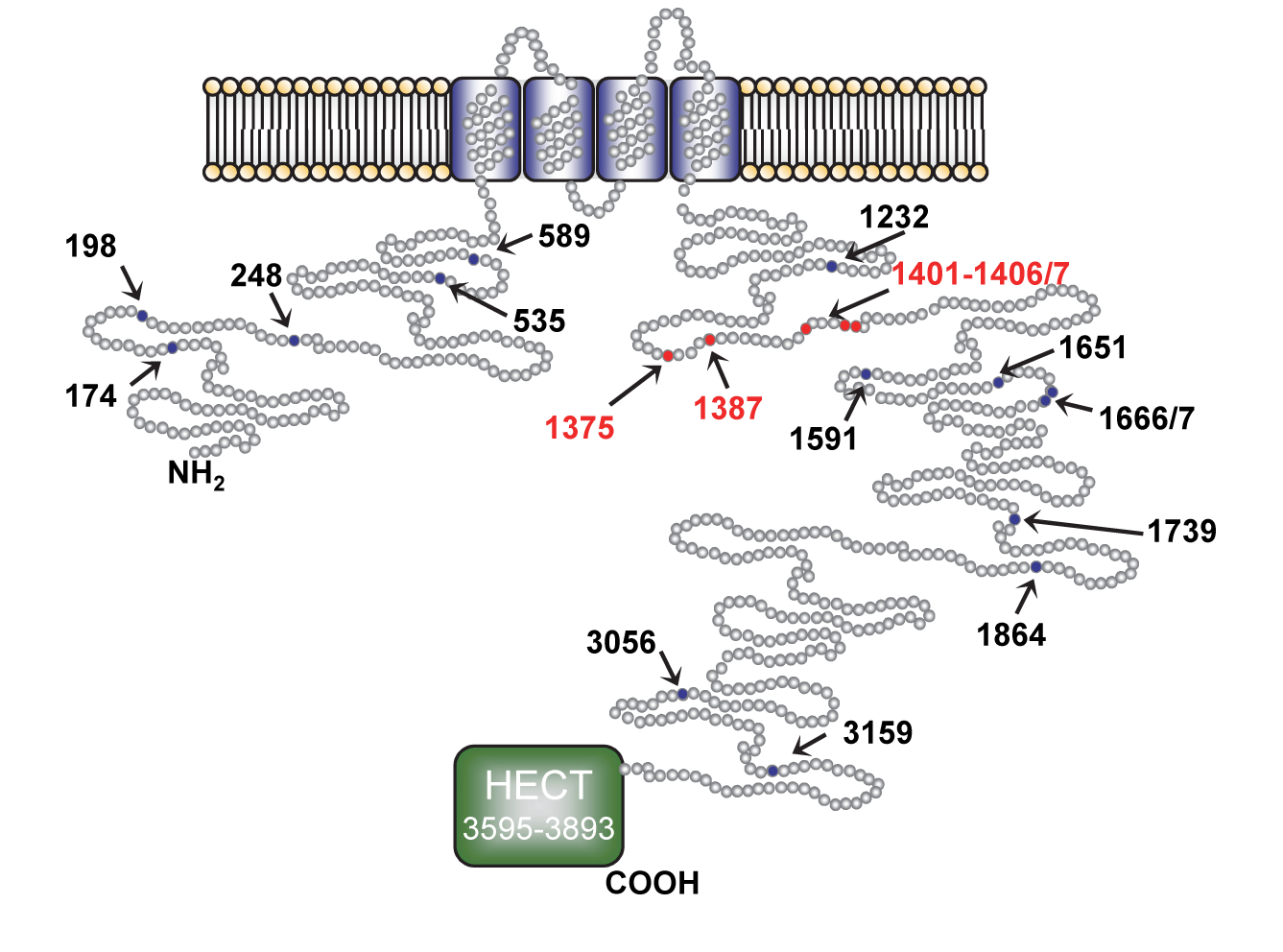


**HECT**

**3595-3893**

**Y347**

**p**

**T372**

**p**

**p**

**S411**

**S1873**

**p**

**K2929**

**Ac**

***piggyBac***

**3334**

***piggyBac***

**1742**

***piggyBac***

**1529**

**Supplementary Figure 1. Topological model of the HECT E3 ubiquitin-protein ligase PfUT.** PfUT is a transmembrane protein consisting of 3893 amino acids and localized at the parasite’s ER/Golgi complex. PfUT contains a C-terminal catalytically-active HECT domain responsible for ubiquitination[^1^](#_ENREF_1). PfUT is phosphorylated at Y347, T372, S411 and S1873 (green) and acetylated at K2929 (purple)[^2^](#_ENREF_2)^,^[^3^](#_ENREF_3). Black arrows point at polymorphic residues associated with quinine responsiveness[^1^](#_ENREF_1). Red colour denotes polymorphisms conserved among different field isolates and laboratory strains, despite distinct geographic origins. Green “p” and blue “Ac” indicate a phosphoryl and an acetyl group, respectively. Blue coloured arrows indicate the three *piggyBac* insertion sites reported by Zhang et al. (2018)[^4^](#_ENREF_4). Adapted from Sanchez et al. (2014)[^1^](#_ENREF_1).

**Supplementary Figure 2. Effect of *pfut* knockdown on *pfcrt* expression in *P. falciparum*. (a)** Western blot analysis of total protein lysates from 3D7 and the *pfut* mutants indicated in the presence and absence of 5 mM GlcN. Blots were probed with an anti-PfCRT antiserum (guinea pig, 1:1000) and an anti-α-tubulin antiserum (mouse, 1:1000). Size standards are indicated in kDa. **(b)** Quantification of Western blots. The intensities of the signals obtained in the Western blots were quantified and the data obtained for PfCRT were normalized against α-tubulin and then expressed in reference to the 3D7 value in the absence of GlcN. Each symbol represents an independent biological replicate. A box plot analysis is overlaid over the individual data points, with the median (thin grey line), mean (thick black line) and the 25% and 75% quartile ranges being shown. Statistical significance was assessed using Holm-Sidak one way ANOVA. n.s. – not significant. This figure was reproduced from the PhD thesis by Jankowska-Döllken^[5](#_ENREF_5)^.

n.s.

41

55

**anti-PfCRT**

**anti-α-tubulin**

**3D7**

**5G**

**6E**

***pfut* mutants**

GlcN

**11B**


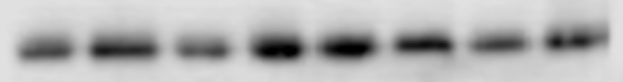

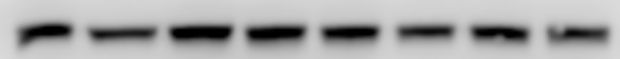


**a**

**b**

**+**

**̶**

**+**

**̶**

**+**

**̶**

**+**

**̶**

kDa


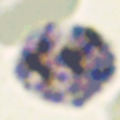


*pfut* mutants

3-7 h

20-24 h

36-40 h

40-44 h

44-48 h

48-52 h

52-56 h

56-60 h


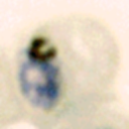

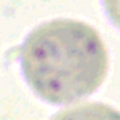

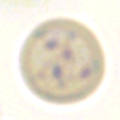

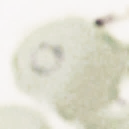

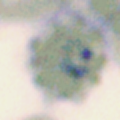

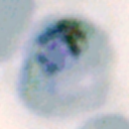

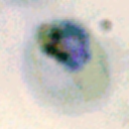

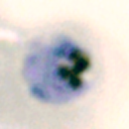

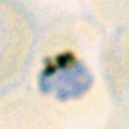

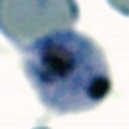

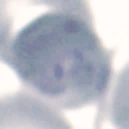

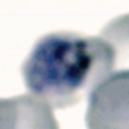

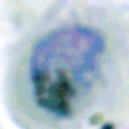

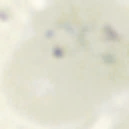

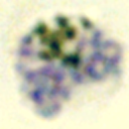

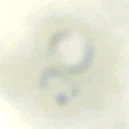

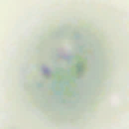

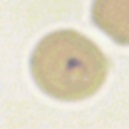

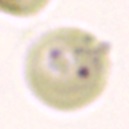

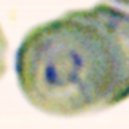

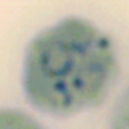

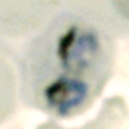

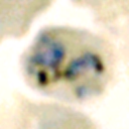

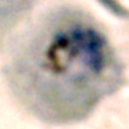

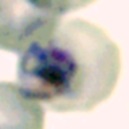

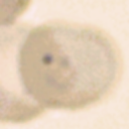

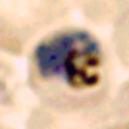

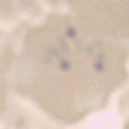

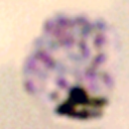

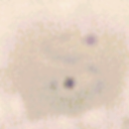

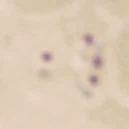


3D7

−

̶+

+

−

GlcN


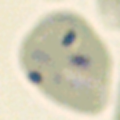

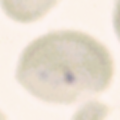

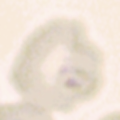

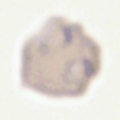

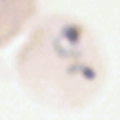

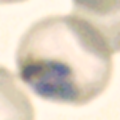

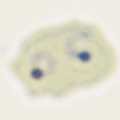

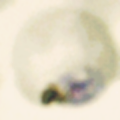

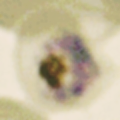

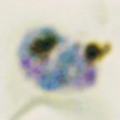

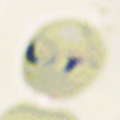

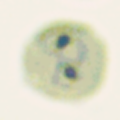


3-7 h

22-26 h

34-38 h

42-46 h

44-48 h

48-52 h

52-56 h

58-62 h


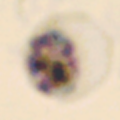

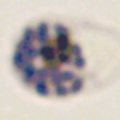

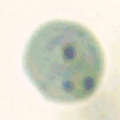

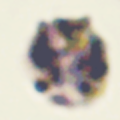


−

+

*pfut* mutants

3D7

*pfut* mutants

3D7

1st cycle

3rd cycle

**Supplementary Figure 3. Morphological analysis.** The Morphology of *pfut* mutants and 3D7 in the presence (48 hours and 144 hours) and absence of GlcN, as determined by Giemsa-stained thin blood smears. The time post infection is indicated. Scale bar: 2 µm. This figure was reproduced from the PhD thesis by Jankowska-Döllken^[5](#_ENREF_5)^.


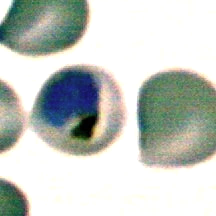

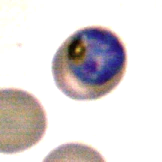

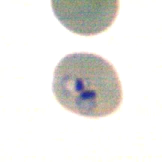

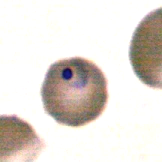

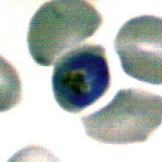

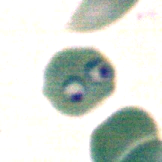

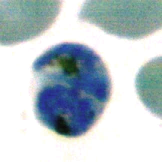

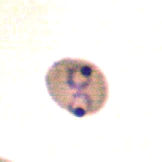

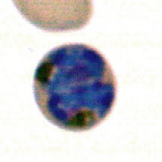

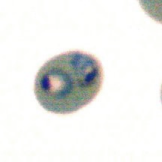

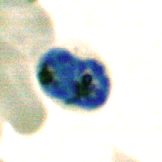

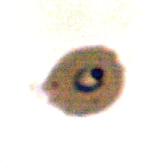

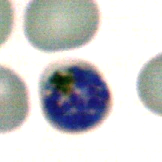

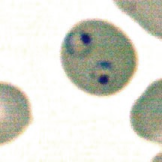

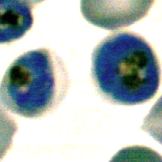


GlcN:

Ø

1.25 mM

2.5 mM

5 mM

0h

24h

48h

72h


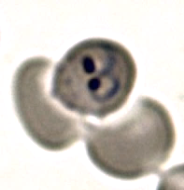

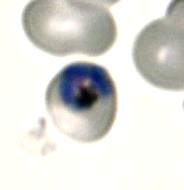

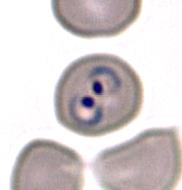

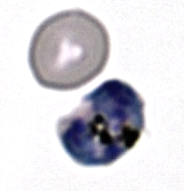


7 mM


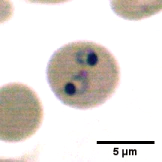


**b**

**a**

**Supplementary Figure 4. Effect of GlcN on growth and morphology of the *P. falciparum* line 3D7. (a)** Growth of 3D7 as a function of different GlcN concentrations. Two representative experiments are shown. Parasitemia was determined from Giemsa-stained thin blood smears. **(b)** Giemsa-stained thin blood smears of parasites treated with different GlcN concentrations for 72 h. At least 1000 parasites were counted per condition. Scale bar: 5 µm. This figure was reproduced from the PhD thesis by Jankowska-Döllken^[5](#_ENREF_5)^.

~~
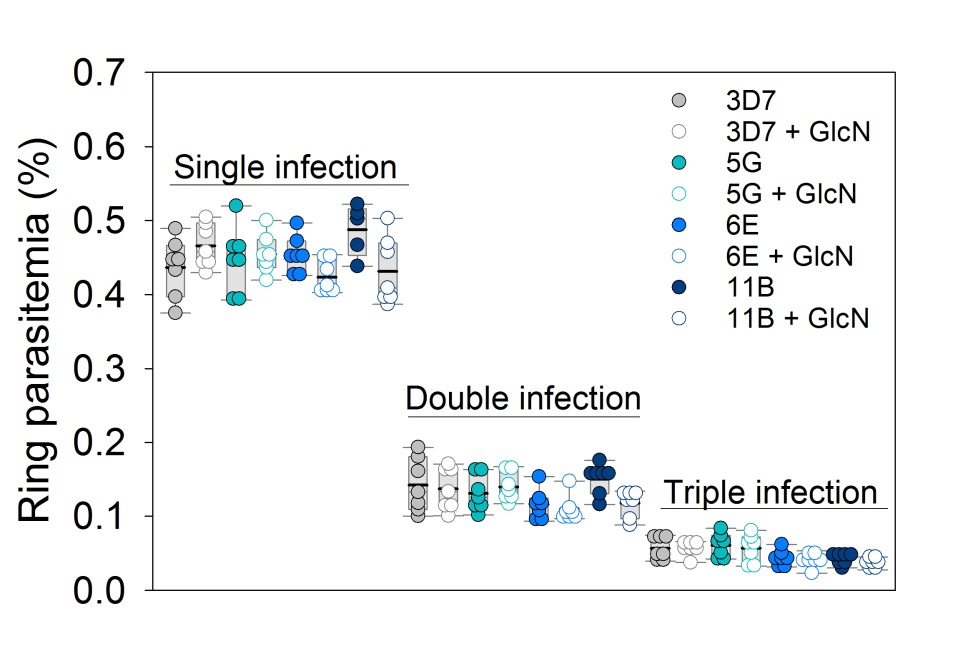
~~

**Supplementary Figure 5. Multiplicity of infection.** The proportion of single-, double- and triple-infected red blood cells was determined in relation to the total ring-stage parasitemia. Each symbol represents an independent biological replicate. A box plot analysis is overlaid over the individual data points, with the median (thin grey line), mean (thick black line) and the 25% and 75% quartile ranges being shown. Statistical significance was assessed using Holm-Sidak one way ANOVA. This figure was reproduced from the PhD thesis by Jankowska-Döllken^[5](#_ENREF_5)^.

y = -3.441x + 27.72

R² = 0.959

Efficiency : **1.95**

y = -3.304x + 25.49

R² = 0.996

Efficiency : **2.01**

**Supplementary Figure 6. Efficiency of the RT-qPCR primer pairs.** A series of 2-fold cDNA dilutions were amplified using primer pairs specific for *pfut* and the housekeeping gene β-tubulin (supplementary Table 1). Results were plotted as mean Ct values of triplicates (mean ± SD) against the log of the cDNA input. A linear regression was subsequently fit to the data points. Primer efficiencies of 1.95 for *pfut* and 2.01 for β-tubulin were obtained from the slopes, indicating a qPCR amplification efficiency of 98% and 100%, respectively. This figure was reproduced from the PhD thesis by Jankowska-Döllken^[5](#_ENREF_5)^.

~~
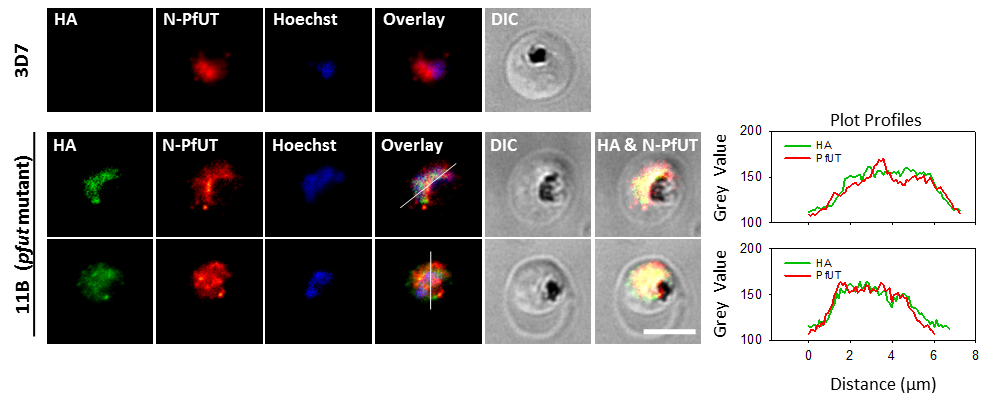
~~

**Supplementary Figure 7. Co-localization of anti-HA and anti-PfUT primary antibodies by indirect immunofluorescence assay (IFA).** Late stage *P. falciparum*-infected erythrocytes were fixed and labelled with anti-HA (mouse, 1:1000) and anti-PfUT antisera (rabbit, 1:1000). Secondary antibody staining was carried out using anti-mouse Alexa Fluor 488 (green) and anti-rabbit Alexa Fluor 546 antibodies (red). The nuclei were visualized with Hoechst (blue). Overlay of the green, red and blue channels is presented. In addition, the differential interference contrast (DIC) and a merged image of the green and red channels and the DIC image are shown. The plot profiles are included for a better presentation of signal co-localization. 3D7 (top panel) lacking a HA-tag served as a control for specificity of anti-HA staining in *pfut* mutants. Scale bar: 5 µm. This figure was reproduced from the PhD thesis by Jankowska-Döllken^[5](#_ENREF_5)^.


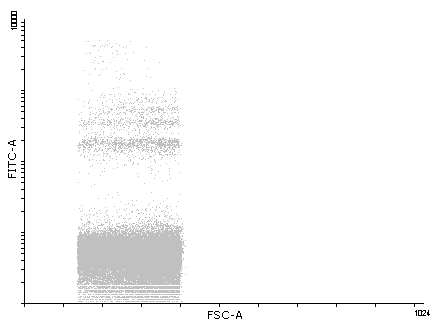


S

R/T

uRBC

Single infection

Double infection

Triple infection

FITC

FSC

**a**


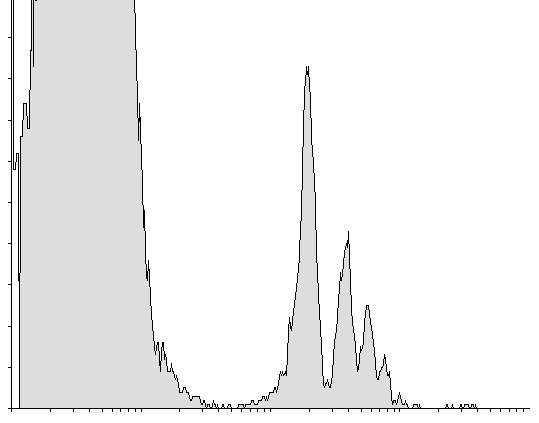


**b**

uRBC

Single

Double

Triple

R/T

S

FITC

Event count

1000

1024

1000

25

**Supplementary Figure 8. Gating strategy used in flow cytometric analysis to discriminate between different parasite stages and between single and multiple infections.** The FITC channel was used to gate populations of uninfected red blood cells (uRBC), ring/trophozoite-stage parasites (R/T) and schizonts (S). **(a)** Dot plot. **(b)** Histogram. This figure was reproduced from the PhD thesis by Jankowska-Döllken^[5](#_ENREF_5)^.

**
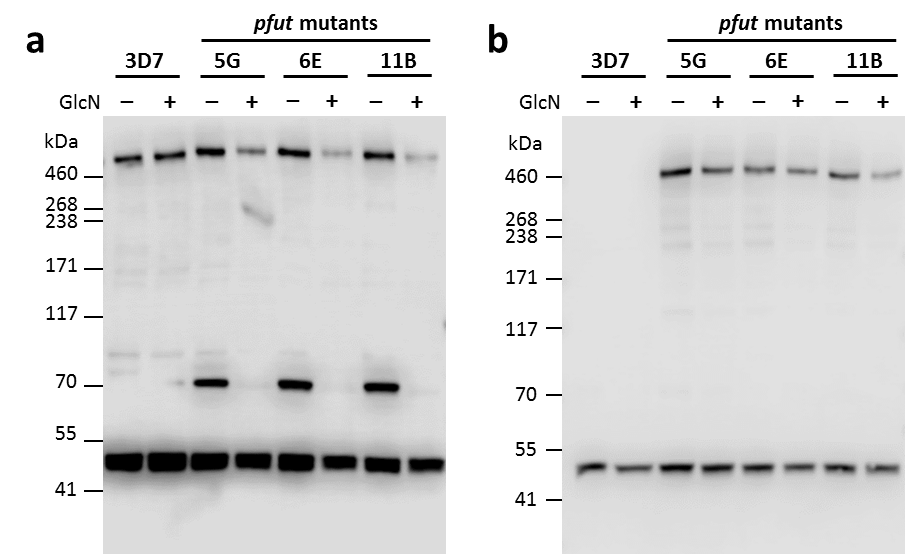
**

**Supplementary Figure 9. Western blot analysis of *pfut* mutants and 3D7**. Full, uncropped views of the blots depicted in Figure 3. **(a)** Top bands, labelling with anti-PfUT antibody raised against residues 473 to 712 of the N-terminal domain[^1^](#_ENREF_1); bottom bands, labelling with anti-α-tubulin antibody. The extra band at 70 kDa displayed by *pfut* mutants in the absence of GlcN is a degradation product containing the N-terminal domain of PfUT. **(b)** Top bands, labelling with anti-HA antibody; bottom bands, labelling with anti-α-tubulin antibody. This figure was reproduced from the PhD thesis by Jankowska-Döllken[^5^](#_ENREF_5).

**References**

1. Sanchez C. P. *et al.* A HECT ubiquitin-protein ligase as a novel candidate gene for altered quinine and quinidine responses in *Plasmodium falciparum*. *PLoS Genet* **10**, e1004382 (2014).

2. Pease B. N. *et al.* Global analysis of protein expression and phosphorylation of three stages of *Plasmodium falciparum* iIntraerythrocytic development. *J Proteome Res* **12**, 4028-4045 (2013).

3. Cobbold S. A., Santos J. M., Ochoa A., Perlman D. H. & Llinás M. Proteome-wide analysis reveals widespread lysine acetylation of major protein complexes in the malaria parasite. *Sci Rep* **6**, 19722 (2016).

4. Zhang M. *et al.* Uncovering the essential genes of the human malaria parasite *Plasmodium falciparu*m by saturation mutagenesis. *Science* **360**, eaap7847 (2018).

5. Jankowska-Döllken M. Functional studies on the chloroquine resistance transporter (PfCRT) and the HECT E3 ubiquitin-protein ligase (PfUT) in *Plasmodium falciparum*. PhD thesis. (Heidelberg University, Heidelberg, Germany, 2019)
